# Predictive Value of Serum Cytokeratin 19 Level for the Feasibility of Conserving Ovaries in Endometrial Cancer

**DOI:** 10.3389/fmed.2021.670109

**Published:** 2021-08-05

**Authors:** Jie Xu, Can Chen, Jing Xiong, Hui Wang, Hua Linghu

**Affiliations:** ^1^Department of Obstetrics and Gynecology, the First Affiliated Hospital of Chongqing Medical University, Chongqing, China; ^2^The First Clinical College, Chongqing Medical University, Chongqing, China; ^3^Department of Obstetrics and Gynecology, Chengdu Women and Children's Central Hospital, Chengdu, China; ^4^School of Medicine, University of Electronic Science and Technology of China, Chengdu, China; ^5^Department of Obstetrics and Gynecology, Chongqing Health Center for Women and Children, Chongqing, China

**Keywords:** pre-operative biomarkers, CK19, ovarian conservation, prognostic factors, endometrial cancer

## Abstract

**Objective:** To determine the predictive value of cytokeratin 19 (CK19) for evaluating the safety of ovarian preservation in patients with endometrial cancer (EC).

**Methods:** Five hundred and seventeen EC patients hospitalized from November 2010 to June 2016 were reviewed retrospectively. Pre-operative tumor biomarkers including CA125, HE4, CK19, and CA19-9 were obtained. Predictive biomarkers associated with ovarian metastasis were selected using univariate and multivariate Logistic regression. The cut-off values were determined by receiver operating characteristic (ROC) curves. Kaplan-Meier method and Cox multivariate regression model was used to perform survival analysis.

**Results:** Among clinical parameters and biomarkers included, age > 65, type II EC, CA125 ≥ 35 u/ml, CK19 > 3.3 ng/ml, and myometrial invasion ≥ 50% depth appeared as significant predictors of the risk of ovarian involvement in univariable logistic analysis. In multivariable analysis, CK19 > 3.3 ng/ml (OR = 11.541, 95%CI: 1.968–67.668, *P* = 0.007) and Type II EC (OR = 8.336, 95%CI: 1.456–47.722, *P* = 0.017) were independent risk predictors of ovarian metastasis in pre-menopausal women. In pre-menopausal women with Type I EC (*n* = 142), CK19 level could satisfactorily predict the risk of ovarian metastasis (AUC = 0.860, 95%CI: 0.792–0.912, *P* < 0.001), and when the cut-off point was set as 2.45 ng/ml, the negative predictive value and negative likelihood ratio were 99% and 0.19, with the maximum Youden index of 0.598.

**Conclusions:** The present study advocates the necessity of incorporating serum CK19 measurement into the pre-operative evaluation of EC, especially as extension of current standard approach with ovarian preservation counseling.

## Introduction

EC is one of the most common cancers in women, the incidence of which ranked first among gynecological malignancies in western counties. In China, an increasing trend of EC incidence has been observed in the 32 Chinese selected cancer registering areas (1). The majority of EC is Type I, namely estrogen-dependent type, which are related to obesity and hyper-estrogenemia (2). Type II EC is non-estrogen-dependent and associated with poorer prognosis compared with type I. The main treatment modality of EC is surgery, including hysterectomy and bilateral salpingo-oophorectomy (BSO). As the majority of EC are estrogen-dependent, the oophorectomy can eliminate the source of estrogen and prevent synchronous and metachronous ovarian cancers. However, pre-menopausal patients will probably undergo an abrupt disruption in ovarian hormone levels following oophorectomy, presenting with series of discomforts. Therefore, the necessity and feasibility of ovary conservation have been under debate.

Some observational studies have revealed that ovarian preservation could be safe for young women with early-stage and low grade EC (3), while others proposed that, for those with no extra-uterine metastasis, poor differentiation or deep myometrial invasion, the normal appearing ovaries of pre-menopausal patients with EC could be preserved (4). Although the preservation could be considered for early stage patients, a possibility ranged 15–20% of recurrence has still been reported (5). Consequently, the identification of these risk factors during or even before surgery and the recognition of microscopic ovarian metastasis during surgery are of great value.

Twenty-five percentage of coexisting ovarian malignancies could be found in women with type I EC aged 45 years or younger (6), and those malignancies that could be recognized during surgery and ovarian micro-metastasis account for only small part (4). For those patients with no extrauterine metastasis, poor differentiation or deep myometrial invasion, it was feasible to do preservation of normal appearing ovaries (4), but the identification of those characteristics could not be well-determined until post-operative histological examination. For this reason, it is important to seek some indicators that could pre-operatively predict the risk of ovarian involvement.

Thus far, there has been no universal consensus on biomarkers for detecting ovarian metastasis in endometrial cancer. Carbohydrate antigen 125 (CA125), the most frequently used biomarker for ovarian cancer, has been employed to predict the synchronous risk factor or metachronous ovarian cancer but with low sensitivity and specificity in diagnosis of EC (7). Human epididymis protein (HE4), a secreted glycoprotein overexpressed by serous and endometrioid epithelial cancer, has a high negative value alone in early detection of ovarian cancer and an improved prediction with combination of CA125 (8). Cyfra21-1, a soluble fragment of cytokeratin19 (CK19) released by cancer cells into circulation (9), plays an important role in diagnosing, predicting and monitoring response to treatment in many kinds of malignancies including non-small cell lung cancer (NSCLC), nasopharyngeal carcinoma, oral/oropharyngeal squamous cell carcinoma, and diagnosis of human papillomavirus infection (10). Interestingly, elevated CK19 level is observed in many patients with EC. Considering the limitations of the existing tumor markers, in present study, we introduce CK19, determine its efficiency in predicting ovarian metastasis and examine its correlation with clinicopathological characteristics.

## Materials and Methods

### Patients and Follow-Up

Patients with EC treated between November 2010 and June 2016 in the Department of Obstetrics and Gynecology, the First Affiliated Hospital of Chongqing Medical University were retrospectively enrolled for this study. Patients were included if they underwent hysterectomy and salpingectomy and were confirmed diagnosis of EC by post-operative pathological reports. Patients without pre-operative pathological specimens before surgical staging or patients with previous history of other malignancies were excluded from the study. Informed consents were obtained from all participants or their families. This study was conducted in accordance with Declaration of Helsinki. The studies involving human participants were reviewed and approved by the Ethics Committee of the First Affiliated Hospital of Chongqing Medical University.

After the initial management, out-patient visit and telephone inquiry took place very 3–6 months since May 2017. The evaluation content contains: patients' general condition, gynecological examination, serum tumor biomarkers assessment, imaging data analysis (sonographic scanning or computed tomography (CT) or magnetic resonance imaging (MRI), and survival status. Overall survival (OS) was defined as the time between diagnosis and the date of death resulting from EC or last follow-up date. Progression free survival (PFS) was defined as the time between diagnosis and the date of the first recurrence or last follow-up date, and the recurrence was determined based on imaging data or histological findings.

### Biomarker Analysis

Within 1 week before surgery, 2–5 ml serum specimen was collected from each patient to detect serum CA125 and HE4 levels by chemiluminescence approach (Abbott Laboratories, US), and serum CA19-9 and CK19 levels by electrochemiluminescence method (Roche cobas e 602, Germany). The normal reference value was as follows: CA125 <35 u/ml, CA19-9 ≤ 27 u/ml, CK19 ≤ 3.3 ng/ml, pre-menopausal HE4 ≤ 70 pmol/L. The ROMA index was calculated from CA125 and HE4 using ovarian cancer risk assessment software (Fujirebio Diagnostics, Japan).

### Statistical Analysis

The data were presented as percentage. Univariate and multivariate logistic regression were used to seek contributing biomarkers for ovarian metastasis. Receiver operating characteristic curves (ROC) were used to compare the ability of these biomarkers to identify patients with ovarian metastasis. The correlation of clinicopathological characteristics and indicated parameters was analyzed by Chi-Squared tests. Survival analysis was performed using Kaplan–Meier (K-M) and Cox multivariate regression analyses, and log-rank test was used to assess differences in survival rates. SPSS 19.0 (IBM, US) was utilized for statistical analysis. The hypothesis was two-tailed and *p* < 0.05 was considered statistically significant.

## Results

Five hundred and seventeen patients with EC underwent hysterectomy between November 2010 and June 2016. Among those, 13 patients who did not undergo bilateral salpingo-oophorectomy were excluded from the study. From total 504 eligible patients, 460 cases had serum CK19 levels measured before surgical treatment. Demographic data and serum biomarker levels are shown in Table 1. The majority of patients were younger than 65 years old (89.6%) with body mass index (BMI) ≤ 30 kg/m^2^ (92.2%), with type I EC (60.4%) and stage I/II diseases (87.0%) [The International Federation of Gynecology and Obstetrics (FIGO) 2009]. Deep myometrial invasion (≥50%) was observed in approximately one-fifth of tumors (22.2%), while the tumor size with 2 cm or larger accounted for 59.3%. Furthermore, elevated CA125 (≥35 u/mL), CA19-9 (>27 u/mL), and CK19 level (>3.3 ng/mL) were found in 24.6, 25.4, and 18.3%, respectively.

**Table 1:** Demographics of patients (*n* = 460).

**Demographic**	**value**
**Age at diagnosis (y)**	
≤65	412 (89.6%)
>65	48 (10.4%)
**BMI (kg/m** ^**2**^ **)**	
≤30	424 (92.2%)
>30	35 (7.6%)
Missing	1 (0.2%)
**CA125**	
<35 u/ml	345 (75.0%)
≥35 u/ml	113 (24.6%)
Missing	2 (0.4%)
**CA19-9**	
≤27 u/ml	343 (74.6%)
>27 u/ml	117 (25.4%)
**CK19**	
≤3.3 ng/ml	376 (81.7%)
>3.3 ng/ml	84 (18.3%)
**Myometrial invasion**	
<50% depth	357 (77.6%)
≥50% depth	102 (22.2%)
Unknown	1 (0.2%)
**Tumor size**	
<2 cm	187 (40.7%)
≥2 cm	273 (59.3%)
**Ovarian metastasis**	
Yes	33 (7.2%)
No	427 (92.8%)
**Type**	
I	278 (60.4%)
II	172 (37.4%)
Unknown	10 (2.2%)
**FIGO 2009**	
I and II	400 (87.0%)
III and IV	60 (13.0%)
**Recurrence**	38
**Death**	21
**Lost to follow-up**	58
**Median follow-up time**	20 months (1–60)

*BMI, body mass index*.

Univariate logistic analysis in total number of patients demonstrated that age > 65 years (*p* = 0.042), CA125 ≥ 35 u/mL (*p* < 0.001), CK19 > 3.3 ng/ml (*p* < 0.001), CA19-9 > 27 u/ml (*p* < 0.001), myometrial invasion >50% (*p* = 0.012) and type II cancer (*p* = 0.001) were all associated with ovarian metastasis (Supplementary Table 1). Six covariates entered the initial equation and adopting the logistic forward step method (entrance 0.05 and deletion 0.10) to construct the model. Finally, three covariates formed the final multivariate model. In multivariate analysis, CA125 ≥ 35 u/mL (*p* < 0.001), CK19 > 3.3 ng/ml (*p* < 0.001) and type II cancer (*p* = 0.008) were testified to be independent risk factors to predict ovarian metastasis (Table 2).

**Table 2:** Logistic multivariate analysis of ovarian metastasis and relevant risk factors in total number of patients.

**Risk factors**	***P-*value**	**OR (odds ratio)**	**95% confidence interval**
CA125	**<0.001**		
<35 u/ml		1	
≥35 u/ml		12.980	5.178–32.538
CK19	**<0.001**		
≤3.3 ng/ml		1	
>3.3 ng/ml		4.963	2.141–11.503
Type	**0.008**		
I		1	
II		3.235	1.361–7.691

*Bold values indicate P-value are statistically significant (P-value < 0.05)*.

Referring to the limited significance of CA125 alone in epithelial ovarian cancer, we brought the data of HE4 and ROMA% value to our stratified analysis for pre-menopausal patients, respectively. In pre-menopausal subgroup analysis, CK19 > 3.3 ng/ml (*p* = 0.007, OR = 11.541, 95%CI 1.968–67.668), type II cancer (*p* = 0.017, OR = 8.336, 95%CI 1.456–47.722) and ROMA% value (*p* = 0.001, OR = 0.955, 95%CI 0.929–0983) were testified to be independent risk factors for ovarian metastasis, both through univariate (Supplementary Table 2) and multivariate analysis (Table 3). Using logistic forward step method to construct the best model, ROMA% instead of CA125 and HE4 entering in the model indicated that the contribution of ROMA% to ovarian metastasis was greater than that of CA125 and HE4 alone.

**Table 3:** Pre-menopausal logistic multivariate analysis of ovarian metastasis and relevant risk factors.

**Risk factors**	***P*-value**	**OR (odds ratio)**	**95% confidence interval**
CK19	**0.007**		
≤3.3 ng/ml		1	
>3.3 ng/ml		11.541	1.968–67.668
Type	**0.017**		
I		1	
II		8.336	1.456–47.722
ROMA%	**0.001**	0.955	0.929–0.983

*Bold values indicate P-value are statistically significant (P-value < 0.05)*.

**Figure 1:**
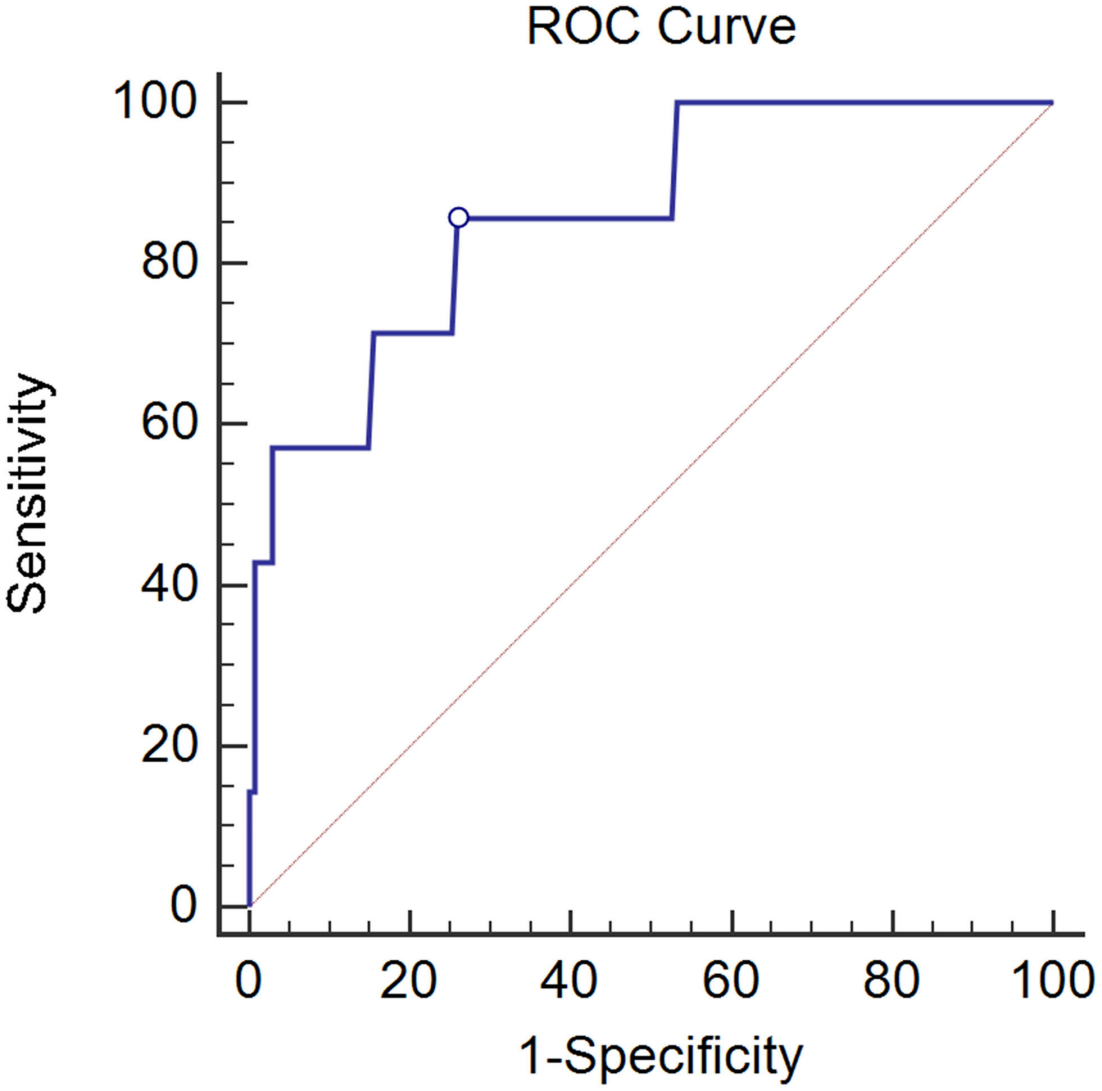
The AUC of CK19 predicting ovarian metastasis in pre-menopausal patients with Type I cancer (*n* = 142). AUC = 0.860, 95% CI 0.792–0.912, *p* < 0.001.

Given the fact that ovarian conserving was mainly required by pre-menopausal patients, among whom type I histology accounted for the majority of cases (11), we analyzed the significance of CK19 in pre-menopausal patients with Type I cancer (*n* = 142) and found ovarian metastasis was higher in cases with CK19 > 3.3 ng/ml compared to those with CK19 ≤ 3.3 ng/ml (26.7 vs. 2.4%, *p* < 0.001). Furthermore, by ROC analysis, CK19 could well predict the risk of ovarian metastasis in pre-menopausal women with Type I cancer (AUC = 0.860, 95%CI 0.792–0.912, *p* < 0.001). In our study, ovarian metastasis occurred in a total of 14 pre-menopausal patients, 7 cases of whom are type I cancer. When the cut-off point was set as 3.3 ng/ml, the sensitivity, specificity, positive and negative predictive value of CK19 in predicting the risk of ovarian metastasis were 57.2, 91.8, 26.7, and 97.6%, respectively. Moreover, when the CK19 cut-off point was set as 2.45 ng/ml, the sensitivity, specificity, positive and negative predictive value, negative likelihood ratio were 85.7, 74.1, 14.6, 99%, and 0.19, with the maximum Youden index of 0.598 (Figure 1).

**Figure 2:**
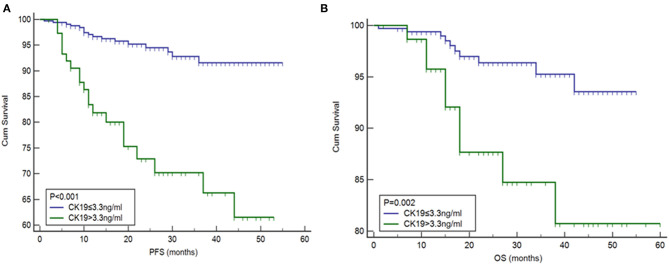
Association between high levels of CK19 with **(A)** progression-free survival (PFS) and **(B)** overall survival (OS).

The correlation between pre-operative CK19 level and clinical characteristics is shown in Table 4. Significant association of higher CK19 level (>3.3 ng/ml) with menopausal (*p* = 0.004), deep myometrial invasion (*P* < 0.001), lymphovascular space invasion (*p* = 0.001), ovarian metastasis (*P* < 0.001), Type II cancer (*p* < 0.001) and advanced FIGO stage (*P* < 0.001) were observed. Follow-up data of 460 patients were lasted until May 2017. Median follow-up time was 20 months with a range from 1 to 60 months. Briefly, 38 cases recurred, 21 patients died, and 58 cases were lost during the follow up. Higher CK19 level (>3.3 ng/ml) exhibited a shorter PFS (Figure 2A), which was further testified to be independent predictor by Cox multivariate regression analysis, as well as deep myometrial invasion and ovarian metastasis (Supplementary Table 3). Similarly, the predictive role of higher CK19 for shorter OS was supported through K-M analysis (Figure 2B). However, elder age (>65 years old), deep myometrial invasion, type II cancer and ovarian metastasis, instead of higher CK19 level, were testified to be independent predictors for OS by Cox multivariate regression analysis (Supplementary Table 4).

**Table 4:** Correlation between pre-treatment CK19 > 3.3 ng/ml and clinical characteristics.

**Demographic**	**Number (*n* = 460)**	**CK19 (ng/ml)**		***P-*value**
		**≤3.3**	**>3.3**	
Age at diagnosis (y)				0.039
≤ 65	412	342	70	
>65	48	34	14	
BMI (kg/m^2^)				0.386
≤ 30	424	344	80	
>30	35	31	4	
Missing	1			
Menopausal				**0.004**
Yes	270	209	61	
No	190	167	23	
Myometrial invasion				**<0.001**
<50% depth	357	304	53	
≥50% depth	102	71	31	
Unknown	1			
Tumor size				0.131
<2 cm	187	159	28	
≥2 cm	273	217	56	
LVSI				**0.001**
Yes	29	17	12	
No	431	359	72	
Ovarian metastasis				<0.001
Yes	33	15	18	
No	427	361	66	
Type				**<0.001**
I	278	244	34	
II	172	123	49	
Unknown	10			
FIGO				**<0.001**
I and II	400	342	58	
III and IV	60	34	26	

*LVSI, lymphovascular space invasion. Bold values indicate P-value are statistically significant (P-value < 0.05)*.

## Discussion

The key finding of this study was that, incorporating serum measurement of CK19 into pre-treatment evaluation may be valuable in EC, especially for young women with type I endometrial cancer who suffer a lot from peri-menopausal syndrome and female hormone supplementation triggered by oophorectomy.

National Comprehensive Cancer Network (NCCN) suggests oophorectomy together with hysterectomy, since this procedure can prevent synchronous and metachronous ovarian cancers. However, such standard treatment may pose challenges to young patients, especially those with desire to keep their fertility. The majority of young patients still opts for oophorectomy due to patients and physicians' concerns of ovarian involvement, although ovarian preservation has not been shown to have association with cancer-related mortality (12) especially in early stage of EC (13), and better OS has been observed among the cases with ovarian conservation compared to oophorectomy cases (14). Consequently, most of young patients with EC, among whom were more likely to die from cardiovascular disease rather than endometrial cancer itself (15), suffer from oophorectomy-followed severe menopausal symptoms and long-term side effects including heart disease, osteoporosis, and nervous system disease. Thus, pre-operative prediction of ovarian metastasis is of great importance for reduction of unnecessary ovarian resection, and prediction of biomarkers is one of great options as it can be obtained before surgery.

Since elevated CK19 level was observed in many patients with EC, we conducted this retrospective study to examine the significance of CK19. The major finding of this study was the predictive role of CK19 for ovarian metastasis, which was verified both through univariate and multivariate analysis as well as in subgroup of type I pre-menopausal EC patients who are the target people of fertility conserving and severe menopausal symptoms suffering. But certainly, the morphologic appearance of the ovaries needs to be evaluated during surgery and removed in case of any abnormalities observed regardless of biomarker levels as the metastasis chance of grossly normal ovaries is low. Besides the significant correlations with elder age, menopausal status, deep myometrial invasion, lympho-vascular space invasion, ovarian metastasis, type II cancer and the advanced FIGO stage, the elevated CK19 level has also independent prognostic value for PFS. Moreover, in present study, the ovarian metastasis was independent prognostic factor for both PFS and OS which means there is still the risk of occult metastasis despite normal ovary appearances. Additionally, eight ovarian micro-metastasis (1.7%) were confirmed by post-operative pathology which could not be recognized during surgery or through pre-operative MRI, including one where normally appearing ovaries preserved during primary surgery found to be involved 6 months later. And surprisingly, in subgroup of pre-menopausal type I EC, we found CK19 level alone can make an satisfactory exclusion diagnosis with its extremely high negative predictive value of 99.0% when cut-off value set as 2.45 ng/ml. Although it would not replace the current standard approach until further verification through large-scale prospective studies, incorporating serum CK19 measurement into pre-treatment evaluation could be useful especially for planned ovarian preservation since it would reduce the risk of unnecessary surgical procedure.

CA125, the most frequently used biomarker in ovarian cancer, has shown to be correlated with (>35 u/mL) an advanced stage EC stage, deep myometrial invasion, positive cytology and lymph node metastases (16). Additionally, FIGO stage I EC patients with occult metastatic disease had a 75% chance of having an elevated serum CA125 level (17). Considering the significance of serum CA125 in epithelial ovarian cancer, we analyzed it in order to evaluate its predictive role in ovarian metastasis in endometrial cancer. Through multivariate logistic analysis in present study, CA125 ≥ 35 u/mL (*P* < 0.001) resulted as an independent risk factor for predicting ovarian metastasis. Although serum CA125 ≥ 35 u/mL was only verified significant in univariable analysis in pre-menopausal subgroup, considering its associations with lymph node metastasis (18) and the extent of disease (19), we recommend that the serum CA125 level should be included in pre-treatment evaluation. HE4, another biomarker that has been shown to aid CA125 in diagnosing and monitoring ovarian cancer and EC, was reported to predict the existence of lymph node metastasis (16), treatment response (20), and prognosis (21) of EC. Although the univariate analysis of our data demonstrated the contributing role of HE4 in predicting ovarian metastasis in pre-menopausal subgroup, the multivariate analysis failed to support this proposition. It is worth mentioning that the ROMA index (calculated by the combination of CA125 and HE4), rather than either CA125 (*P* = 0.141) or HE4 (*P* = 0.757) alone appears more predictive significance for ovarian metastasis in pre-menopause subgroup, and the reason may be that the calculation of ROMA value was stratified by menopause status.

Matsuo et al. have shown that women younger than 40 years of age would be more likely to have ovary (ovaries) conserved (14). Likewise, we supposed that the ovarian conservation would be more valuable for pre-menopausal women, especially those younger than 40. Among all pre-menopausal patients in the present study, no significant correlation was found between elder age (>40 years old) and higher risk of ovarian involvement (*P* = 0.579). These data provided a support for preserving ovaries in pre-menopausal patients with EC, especially in those patients with type I EC with CK19 ≤ 2.45 ng/ml.

Undetermined pre-operative type and histology was found in 206 patients, and 15 cases with pre-operative diagnosis of type I EC were corrected as type II EC after hysterectomy, most probably because of the limited sampling of pre-operative endometrial biopsy (22). The concordance rates of the pre-operative and final pathological report are reported to be varied between 56 and 81% (23). 7.2% had downgrade discordance, and the most common type of downgrade was seen in type I EC (23). Thus, improvement of accuracy of pre-operative pathology should be addressed in the future studies.

The limitations of this study include the nature of the study which was retrospective. Additionally, a significant correlation between elevated CK19 level was found for PFS but not for OS. The possible reasons for this include the limited follow-up period, and the fact that, a subset of patients have to give up further treatment for financial reasons. Furthermore, the cases of co-existed ovarian cancer and endometrial cancer were not included. One of advantages of present study will be the first to report pre-operative prediction value of the biomarker of CK19 for ovarian metastasis in EC, to our knowledge. Second, a satisfactory negative predictive value of 99% of CK19 for ovarian metastasis in subgroup of pre-menopausal type I EC has reached, which demonstrated a perfect exclusive test for there is only 1% possibility of false negative rate when serum CK19 is <2.45 ng/ml. Third, collaborative prediction of multiple biomarkers for ovarian metastasis has built a good predictive model in targeting patients, providing evidence for clinical practice.

In conclusion, the present study advocates the necessity of incorporating serum CK19 measurement into the pre-treatment evaluation of EC, especially as extension of current standard approach with ovarian preservation counseling.

## Data Availability Statement

The datasets generated during the current study are not publicly available but are available from the corresponding author on reasonable request.

## Ethics Statement

The studies involving human participants were reviewed and approved by the Ethics Committee of the First Affiliated Hospital of Chongqing Medical University.

## Author Contributions

JXu, CC, and JXi have collected and analyzed all the data. JXu and CC wrote the primary draft of this manuscript. HW provided some data during out-patient visit and helped with the discussion part. HL proposed, designed and revised the manuscript and is responsible for the reliability and explanation of all the data. All authors contributed to the article and approved the submitted version.

## Conflict of Interest

The authors declare that the research was conducted in the absence of any commercial or financial relationships that could be construed as a potential conflict of interest.

## Publisher's Note

All claims expressed in this article are solely those of the authors and do not necessarily represent those of their affiliated organizations, or those of the publisher, the editors and the reviewers. Any product that may be evaluated in this article, or claim that may be made by its manufacturer, is not guaranteed or endorsed by the publisher.

## Abbreviations

CK19: Cytokeratin 19
EC: Endometrial Cancer
ROC: receiver operator curves
BSO: Bilateral salpingo-oophorectomy
CA125: Carbohydrate antigen 125
CT: computed tomography
MRI: Magnetic Resonance Imaging
PFS: Progression Free Survival
OS: Overall Survival
BMI: body mass index
K-M: Kaplan–Meier
FIGO: The International Federation of Gynecology and Obstetrics
NCCN: National Comprehensive Cancer Network.
