# Supplementary material for: Predictive Value of Serum Cytokeratin 19 Level for the Feasibility of Conserving Ovaries in Endometrial Cancer
Source: Front Med (Lausanne). 2021 Aug 5;8:670109. doi: 10.3389/fmed.2021.670109 (PMC8374735; doi:10.3389/fmed.2021.670109)
Supplement: Supplementary file 1 [file Data_Sheet_1.doc]

**Supplemental Table 1: Logistic univariate analysis of ovarian metastasis and relevant risk factors in total number of patients:**

| **Risk factors** | **P value** | **OR(odds ratio)** | **95% confidence interval** |
| --- | --- | --- | --- |
| Age(years)  ≤65  >65 | **0.042** | 1  2.535 | 1.036-6.200 |
| BMI(kg/m2)  ≤30  >30 | 0.318 | 1  1.758 | 0.581-5.319 |
| CA125  <35u/ml  ≥35u/ml | **<0.001** | 1  13.718 | 5.745-32.753 |
| CK19  ≤3.3ng/ml  >3.3ng/ml | **<0.001** | 1  6.564 | 3.151-13.671 |
| CA19-9  ≤27u/ml  >27u/ml | **<0.001** | 1  4556 | 2.204-9.419 |
| Tumor size  <2cm  ≥2cm | 0.109 | 1  1.906 | 0.865-4.200 |
| Myometrial invasion  <50% depth  ≥50% depth | **0.012** | 1  2.598 | 1.236-5.463 |
| Type  I  II | **0.001** | 1  3.560 | 1.680-7.543 |

Abbreviations: BMI, body mass index

**Supplemental Table 2: Logistic univariate analysis for ovarian metastasis and relevant risk factors in Premenopausal patients**

| **Risk factors** | **P value** | **OR(odds ratio)** | **95% confidence interval** |
| --- | --- | --- | --- |
| Age(years)  ≤40  >40 | 0.579 | 1  1.804 | 0.224-14.534 |
| BMI (kg/m2)  ≤30  >30 | 0.999 | - | - |
| CA125  <35u/ml  ≥35u/ml | **0.001** | 1  10.388 | 2.733-39.479 |
| HE4  ≤70pmol/L  >70pmol/L | **0.003** | 1  8.444 | 2.106-33.867 |
| ROMA% | **<0.001** | 0.956 | 0.935-0.977 |
| CK19  ≤3.3ng/ml  >3.3ng/ml | **<0.001** | 1  11.740 | 3.517-39.188 |
| CA19-9  ≤27u/ml  >27u/ml | **0.001** | 1  8.166 | 2.494-26.737 |
| Tumor size  <2cm  ≥2cm | 0.103 | 1  3.011 | 0.801-11.310 |
| Myometrial invasion  <50% depth  ≥50% depth | 0.251 | 1  2.229 | 0.567-8.755 |
| Type  I  II | **0.023** | 1  3.785 | 1.204-11.896 |

Abbreviations: BMI, body mass index

**Supplemental Table 3: Cox multivariate analyses of progression free survival**

| **Risk factors** | **P value** | **HR(hazard ratio)** | **95% confidence interval** |
| --- | --- | --- | --- |
| Age at diagnosis (y)  ≤65  >65 | **0.145** | 1  2.289 | 0.753-6.963 |
| CA125  <35u/ml  ≥35u/ml | 0.735 | 1  1.189 | 0.436-3.244 |
| CK19  ≤3.3ng/ml  >3.3ng/ml | **0.004** | 1  3.298 | 1.475-7.372 |
| lymph node status  negative  positive | 0.124 | 1  2.513 | 0.777-8.129 |
| Myometrial invasion  <50% depth  ≥50% depth | **<0.001** | 1  6.968 | 2.901-16.737 |
| Ovarian metastasis  No  Yes | 0.032 | 1  3.728 | 1.122-12.390 |
| Type  I  II | 0.117 | 1  2.019 | 0.839-4.856 |
| LVSI  No  Yes | **0.691** | 1  1.294 | 0.363-4.610 |

Abbreviations: LVSI — lymphovascular space invasion

**Supplemental Table 4: Cox multivariate analyses of overall survival**

| **Risk factors** | **P value** | **HR(hazard ratio)** | **95% confidence interval** |
| --- | --- | --- | --- |
| Age at diagnosis (y)  ≤65  >65 | **0.007** | 1  5.653 | 1.602-19.946 |
| CA125  <35u/ml  ≥35u/ml | 0.878 | 1  1.120 | 0.264-4.745 |
| CK19  ≤3.3ng/ml  >3.3ng/ml | 0.624 | 1  1.349 | 0.408-4.464 |
| lymph node status  negative  positive | 0.767 | 1  1.405 | 0.148-13.367 |
| Myometrial invasion  <50% depth  ≥50% depth | 0.002 | 1  6.787 | 2.067-22.279 |
| Ovarian metastasis  No  Yes | 0.011 | 1  9.985 | 1.679-59.363 |
| Type  I  II | **0.058** | 1  3.533 | 0.958-13.032 |
